# Mammography prevalence in Mexico from 2001-2018: Results from the Mexican Health and Aging study

**DOI:** 10.1016/j.pmedr.2023.102150

**Published:** 2023-02-14

**Authors:** Sean P. McClellan, Karla Unger-Saldaña, John M. Neuhaus, Michael B. Potter, Carmen García-Peña, Jacqueline M. Torres

**Affiliations:** a Department of Family and Community Medicine, University of California San Francisco, San Francisco, CA, USA; b National Cancer Institute, Mexico City, Mexico; c Department of Epidemiology and Biostatistics, University of California San Francisco, San Francisco, CA, USA; d National Institute of Geriatrics, Mexico City, Mexico

## Abstract

After introducing guidelines for breast cancer screening in 2003, Mexico began to prioritize the implementation of mammography screening nationally. Since then, there have been no studies assessing changes in mammography in Mexico using the two-year prevalence interval that corresponds to national guidelines for screening frequency. The present study analyzes the Mexican Health and Aging Study (MHAS), a national population-based panel study of adults aged 50 and older, to evaluate changes in 2-year mammography prevalence among women aged 50 to 69 across five survey waves from 2001 to 2018 (n = 11,773). We calculated unadjusted and adjusted mammography prevalence by survey year and health insurance type. Overall prevalence increased substantially from 2003 to 2012 and leveled off in the period from 2012 to 2018 (2001: 20.2 % [95 % CI 18.3, 22.1]; 2003: 22.7 % [20.4, 25.0]; 2012: 56.5 % [53.2, 59.7]; 2015: 62.0 % [58.8, 65.2]; 2018: 59.4 % [56.7,62.1]; unadjusted prevalence). Prevalence was higher among respondents with social security insurance, who are more likely to work in the formal economy, than among respondents without social security, who are more likely to work in the informal economy or be unemployed. The overall prevalence estimates observed were higher than previously published estimates of mammography prevalence in Mexico. More research is needed to confirm findings regarding two-year mammography prevalence in Mexico and to better understand the causes of observed disparities.

## Highlights

• This was the first study of changes in two-year mammography prevalence in Mexico.
• Mammography prevalence increased from 2001 to 2018 in this representative sample.
• Disparities in prevalence related to insurance type persisted.
• Mammography prevalence estimates were higher than in previously published studies.

## 1 Introduction

Breast cancer is the number one cause of cancer death among women globally as well as among women in Mexico (The Global Cancer Observatory, 2021, Sung et al., 2021). The World Health Organization conditionally recommends breast cancer screening in limited resource settings with strong healthcare systems based on moderate quality evidence, while it encourages the prioritization of the early diagnosis of symptomatic breast cancer in limited resource settings with weak healthcare systems (World Health Organization, 2014). Several upper middle income countries have implemented screening mammography programs, however, these programs are not usually implemented in an organized fashion and are not always able to guarantee the quality assurance and access to diagnosis and treatment that are recommended as prerequisites to screening within the international discourse (Ginsburg et al., 2020).

When Mexican guidelines for breast cancer screening were first introduced in 2003 they recommended annual mammography screening for average risk women aged 50–69 and biannual screening for women age 40–49 with risk factors (NOM, 2003). In 2011 these guidelines were updated to recommend biannual mammography screening for all women age 40–69 (NOM, 2011). During the decade following the introduction of breast cancer screening guidelines; the number of mammography machines available in the Mexican healthcare system increased substantially; and from 2007 to 2017 the government implemented two national action plans for breast and cervical cancer that included a goal of increasing participation in screening mammography (Martínez-Montañez et al., 2009, Secretaria de Salud, 2013, Secretaria de Salud, 2012). During this same period; non-governmental organizations in Mexico were also active promoting mammography (Knaul et al., 2009).

There have been few studies assessing changes in mammography prevalence in the period since Mexico adopted guidelines recommending screening. Current estimates of mammography prevalence are derived from cross-sectional studies or repeat cross-sectional studies that span a limited time period (Pengpid et al., 2020, Akinyemiju et al., 2016, Agudelo, 2013). Some studies ask respondents about breast exam and mammography in the same question making results difficult to interpret (Rivera-Hernández et al., 2019). National guidelines recommend mammography every-two years, but few studies ask about the completion of screening in the past two years, making it difficult to assess prevalence according to the intervals recommended by national guidelines (Pagán et al., 2007, Torres-Mejía et al., 2013).

Additionally, more information is needed about differences in screening prevalence between sectors of the Mexican healthcare system. Prior research has shown that use of some preventive services is higher among social security insurance beneficiaries than among persons without social security insurance (Rivera-Hernández et al., 2019, Balandrán-Duarte et al., 2020). Social security beneficiaries mostly work in the formal economy and benefit from higher rates of public healthcare spending than those without social security who are more likely to work in the informal economy or be unemployed (González Block et al., 2020).

*The present study evaluates national changes in mammography prevalence by insurance type from 2001 to 2018 among women in Mexico ages 50 to 69 using national-level survey data. We hypothesize that screening prevalence would increase during the study period, but that there would be persistent disparities in screening prevalence between respondents with and without social security.*

## 2 Methods

### 2.1 Setting

The Mexican health system is divided into three main sectors. The first includes several social security institutions that provide health services for current and retired employees of private companies and their family members (*Instituto Mexicano de Seguro Social,* IMSS), federal and state government organizations (*Instituto de Seguridad y Servicios Sociales de los Trabajadores del Estado,* ISSSTE) and other smaller institutions. The second includes Ministry of Health facilities that are mostly used by the population without social security. The third includes private sector facilities that are used by a small population with private health insurance, but also frequently by the rest of the population (including people with and without social security) (González Block et al., 2020) In 2004, Mexico implemented an insurance program known as *Seguro Popular* for people not covered by social security institutions. By 2018, 43 % of the Mexican population was covered by *Seguro Popular*, 42 % was covered by several social security schemes, and 15 % of the population was not affiliated with either *Seguro Popular* or social security (González Block et al., 2020).

Prior research describing the context of mammography screening in Mexico is limited. Mammography screening in Mexico is opportunistic; there are no large-scale population screening programs. All sectors of the health system include screening mammography as one of the preventive services they offer patients, however, access to screening mammography may be inconsistent due to limited infrastructure and human resources (Uscanga-Sánchez et al., 2014). In addition to health system factors, breast cancer stigma and limited knowledge of breast cancer screening among providers and patients have been identified as barriers to the implementation of mammography screening (Nigenda et al., 2009).

### 2.2 Data source and sample

This study was an analysis of the Mexican Health and Aging Study (MHAS), a national panel study of adults age 50 and older in Mexico with protocols similar to the Health and Retirement Study in the United States (Mexican Health and Aging Study, 2022). A detailed description of the MHAS cohorts and methodology is available elsewhere (Wong et al., 2017, Mexican Health and Aging Study, 2012). The baseline MHAS survey was conducted in 2001 with follow up interviews in 2003, 2012, 2015 and 2018 and new cohorts added to replenish the sample in 2012 and 2018. Response rates ranged from 84.7 % to 93.3 % across survey waves.

The survey employs a stratified clustered sample design. To generate nationally representative cohorts of Mexican adults aged 50 and older, the MHAS employed strata, sampling units and survey weights adjusting for non-response and intentional oversampling from states with historically high migration to the U.S. that were provided by the Nacional de Estadística y Geografía (INEGI). However, due to concerns about participant identification the publicly available datasets used for this study do not contain information on strata or clustering, so we were unable to incorporate these into our analyses. Survey weights are included in the public datasets and were incorporated into all analyses. MHAS is partly sponsored by the National Institutes of Health/National Institute on Aging (Grant No NIH R01AG018016) in the United States and INEGI in Mexico. Data files and documentation are public use and available at https://www.MHASweb.org.

Across all survey waves, the MHAS contained 26,880 respondents who completed an interview including 15,057 women providing 46,726 observations. Of these observations 41,381 were from direct interviews (as opposed to proxy interviews which did not ask about mammography) and 26,765 of these were from women aged 50 to 69 at the time of the interview. We excluded 201 observations where the participant endorsed a personal history of breast cancer as well as 266 observations with missing screening and covariate data. The analytic sample size included 26,177 observations from women aged 50–69 at the time of interview representing 11,773 unique respondents. This project only used publicly available anonymized data and did not require review from the University of California San Francisco institutional review board.

### 2.3 Outcome variable

The study outcome was mammography prevalence within two years prior to the interview date. The survey question asking about mammography is included in Appendix 1. Responses of “Don’t know” or “Refused” were recoded as missing.

### 2.4 Exposure variables

The exposures considered were survey year and respondent insurance type. In regression models used to estimate prevalence, survey year was treated as a categorical variable to allow flexibility when modeling changes in mammography prevalence across waves. In models assessing for linear trends, survey year was treated as a continuous variable. To assess insurance type, interviewers asked respondents “Do you have the right to medical care at…” and then listed options for 1) IMSS 2) ISSSTE 3) social security services affiliated with the national petroleum company or the Mexican armed forces (other social security) 4) Seguro Popular (for 2012, 2015, and 2018 waves only) 5) private medical insurance and 6) other.

Respondents who reported having both social security insurance and another insurance type were categorized according to their social security insurance. Social security insurance tends to provide more comprehensive services and it was thought that for patients with access to multiple systems, the system with the most comprehensive coverage would best predict mammography prevalence (González Block et al., 2020). Respondents who had access to more than one social security system were categorized as ‘multiple social security’. Respondents with any combination of Seguro Popular, private insurance, other insurance or no insurance were grouped together as having no social security.

### 2.5 Covariates

Covariates were selected a priori based on factors known to predict both insurance type and screening mammography. These included continuous age in years, years of education, marital status (single; married/in a civil union; divorced/separated; widowed) and size of the respondent’s locality of residence (greater than 100,000 residents; 15,000 to 99,999; 2,500 to 14,999; less than 2,500). We also included a measure of household goods that considered household ownership of up to six or eight items depending on the survey wave. In waves 2001 and 2003 the items included were a radio, a TV, a refrigerator, a washing machine, a phone, and a heater. In waves 2012 to 2018 computer and internet were added to reflect the changing prevalence of household goods ownership. Prior studies have demonstrated that similar measures of household items were strongly correlated with household income and wealth and were predictive of health trajectories later in life (Wong and Espinoza, 2003, Torres et al., 2018).

### 2.6 Analysis

All analyses incorporated individual survey weights. First, we calculated descriptive statistics stratifying by survey year and then separately by insurance type. Second, we estimated the unadjusted prevalence of mammography completion in the prior two years for each survey wave for the three most common insurance types as well as for all respondents across all insurance types. We also estimated differences in prevalence between consecutive survey waves. As a sensitivity analysis, we estimated unadjusted prevalence including only respondents being interviewed for the first time. To account for repeated observations across survey waves when estimating confidence intervals, we used the survey R package (4.1–1; Lumley 2019) which uses influence functions to estimate the variance and covariance of survey sample statistics (Deville, 1999).

Third, to assess for linear trends in mammography prevalence among all respondents we used Poisson regression fit by generalized estimating equations with an exchangeable correlation structure and robust standard errors. Survey year in continuous years and insurance type were included as predictors as well as a statistically significant interaction term between these two variables. The use of generalized estimating equations with robust standard errors allows for the estimation of population means while accounting for repeat observations across survey waves (Hubbard et al., 2010, Diggle et al., 2002). Linear combinations of coefficients from this interaction model were used to assess for linear trends in mammography prevalence among beneficiaries of the three most common insurance types.

Fourth, we estimated the adjusted prevalence of mammography completion using Poisson regression fit by generalized estimating equations with an exchangeable correlation and robust standard errors. We used this model to estimate adjusted predictions at the mean for mammography prevalence and to test for differences in adjusted prevalence by survey year and insurance type via an interaction term between survey wave and insurance type.

Fifth, we estimated unadjusted and adjusted prevalence differences in mammography completion between the three most common insurance types by year, using the same methods described above to estimate differences. For adjusted prevalence differences, we used the same generalized estimating equation described above.

Finally, we estimated unadjusted prevalence by locality size for each survey wave as well as differences in prevalence between consecutive survey waves. All data analysis was completed using R Statistical Software (4.2.1; 2022 R Core Team).

## 3 Results

Median age and years education increased across survey waves (Table 1). When stratified by insurance type, individuals without social security were more likely to reside in rural areas, report fewer years of education, and own fewer household goods than individuals with social security (Supplementary Table 1).

**Table 1:** Respondent characteristics by survey year for women aged 50–69 in the Mexican Health and Aging Study.

		2001 n = 5170	2003 n = 4885	2012 n = 5507	2015 n = 4925	2018 n = 5690
Age, Median (IQR)		57.0 (53.0,63.0)	59.0 (55.0,63.0)	58.0 (54.0,62.0)	59.0 (55.0,63.0)	57.0 (53.0,62.0)
Education, Median (IQR)		3.0 (0.0,6.0)	3.0 (0.0,6.0)	6.0 (3.0,9.0)	6.0 (3.0,9.0)	6.0 (3.0,9.0)
Household goods^a^, Median (IQR)		4.0 (3.0,6.0)	5.0 (3.0,6.0)	5.0 (4.0,7.0)	6.0 (4.0,7.0)	6.0 (4.0,7.0)
Marital status, %	Single	4.5	4.7	5.8	5.9	6.8
	Married / in a civil union	64.0	62.2	66.0	64.3	68.1
	Divorced / separated	13.0	10.7	14.7	16.7	13.3
	Widowed	18.4	22.4	13.5	13.2	11.8
Locality size, %	100,000+	49.3	48.7	52.2	51.9	47.9
	15,000–99,999	13.7	14.4	15.3	15.8	14.7
	2,500–14,999	12.9	12.6	12.9	12.7	14.6
	less than2,500	24.2	24.3	19.6	19.6	22.8
Insurance type^b^, %	IMSS	40.5	40.5	38.8	41.0	39.7
	ISSSTE	9.2	8.5	10.2	11.8	10.4
	Other, social security	1.5	2.0	2.0	1.4	1.0
	Multiple, social security	3.0	2.7	2.8	3.9	2.4
	No social security^c^	45.9	46.3	46.2	41.8	46.4

Note: Estimated using survey weights. No social security includes respondents with Seguro Popular, no insurance, private insurance and other, no social security. IMSS = Instituto Mexicano de Seguro Social ISSSTE = Instituto de Seguridad y Servicios Sociales de los Trabajadores del Estado.

a. Maximum number of household goods increase from 5 in waves 2001 to 2012 to 7 in waves 2015 to 2018.

b. Some respondents had more than one type of insurance. Respondents with social security and another type of insurance were categorized according to their social security insurance.

c. No social security includes respondents with Seguro Popular, no insurance, private insurance and other, no social security.

**Fig. 1:**
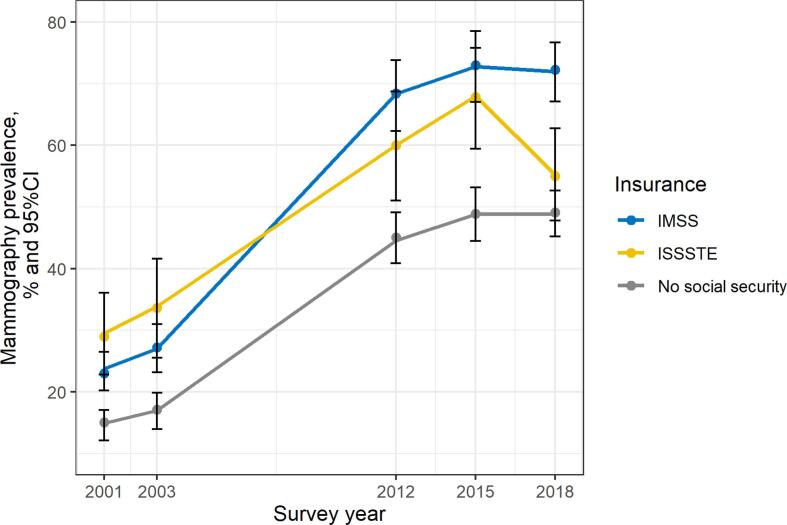
Unadjusted mammography prevalence by survey year for the three most common insurance type among women aged 50–69 in the Mexican Health and Aging Study Note: Estimated using survey weights. IMSS = Instituto Mexicano de Seguro Social ISSSTE = Instituto de Seguridad y Servicios Sociales de los Trabajadores del Estado.

Fig. 1 and Table 2 present unadjusted changes in mammography prevalence for all respondents and for the three most common insurance types. Prevalence estimates for the insurance types other, social security and multiple, social security were not reported as these insurance types were uncommon and corresponding prevalence estimates had low precision. From 2001 to 2003 mammography prevalence did not change substantially overall or for any of the most common insurance types. From 2003 to 2012, there was a marked increase in mammography prevalence overall (33.8 % [95 %CI 29.7 %, 37.8 %]) and for all three of the most common insurance types (IMSS 41.0 % [33.9 %, 48.1 %], ISSSTE 26.3 [14.3 %, 38.3 %], no social security 28.1 % [22.8 %, 33.3 %]). After 2012 this upward tendency in screening prevalence leveled off with only modest increases in mammography prevalence between 2012 and 2015 (overall 5.5 % [−0.1, 11.1]; IMSS 4.7 % [−5.8, 15.3]; ISSSTE 7.7 [−4.0, 19.5]; no social security 3.8 [−3.2, 10.8]). There were no substantial changes in prevalence from 2015 to 2018 overall or among IMSS beneficiaries and respondents without social security, while for ISSSTE beneficiaries’ mammography prevalence decreased from 2015 to 2018 (overall: −2.6 % [−7.6 %, 2.4 %]; IMSS: −0.9 % [−10.7 %, 8.9 %]; ISSSTE: −12.4 % [–23.8 %, −0.9 %]; no social security: 0.1 % [−6.0 %, 6.2 %]).

**Table 2:** Unadjusted mammography prevalence by survey wave for the three most common insurance types and overall, for women aged 50–69 in the Mexican Health and Aging Study, % (95 % CI).

	2001	2003		2012		2015		2018	
Insurance	Prevalence	Difference from prior wave	Prevalence	Difference from prior wave	Prevalence	Difference from prior wave	Prevalence	Difference from prior wave	Prevalence
IMSS	23.4 (20.2, 26.5)	3.7 (−1.7, 9.1)	27.1 (23.2, 30.9)	41.0 (33.9, 48.1)	68.0 (62.3, 73.8)	4.7 (−5.8, 15.3)	72.8 (67.0, 78.5)	−0.9 (−10.7, 8.9)	71.9 (67.1, 76.7)
ISSSTE	29.4 (22.8, 36.0)	4.1 (−6.6, 14.9)	33.6 (25.5, 41.6)	26.3 (14.3, 38.3)	59.8 (51.0, 68.7)	7.7 (−4.0, 19.5)	67.6 (59.4, 75.8)	−12.4 (–23.8, −0.9)	55.2 (47.8, 62.7)
No social security	14.5 (12.1, 17.0)	2.4 (−2.1, 6.9)	16.9 (13.9, 19.9)	28.1 (22.8, 33.3)	45.0 (40.9, 49.1)	3.8 (−3.2, 10.8)	48.8 (44.5, 53.2)	0.1 (−6.0, 6.2)	48.9 (45.2, 52.7)
Overall	20.2 (18.3, 22.1)	2.5 (−0.9, 5.8)	22.7 (20.4, 25.0)	33.8 (29.7, 37.8)	56.5 (53.2, 59.7)	5.5 (−0.1, 11.1)	62.0 (58.8, 65.2)	−2.6 (−7.6, 2.4)	59.4 (56.7, 62.1)

Note: Estimated using survey weights. IMSS = Instituto Mexicano de Seguro Social ISSSTE = Instituto de Seguridad y Servicios Sociales de los Trabajadores del Estado.

Between 2001 and 2018 there were positive linear trends in mammography prevalence among beneficiaries of the three most common insurance types (Supplementary Table 2). These trends varied by insurance type (Wald test for interaction term, p < 0.001). In a sensitivity analysis that only included first time respondents to the survey, unadjusted prevalence estimates did not change substantially (Supplementary Table 3). Adjusted estimates were qualitatively similar (Supplementary Table 4). In the adjusted regression model, there was a statistically significant interaction between survey year and insurance type (Wald test, p =.002) suggesting that changes in mammography prevalence differed by insurance type. Although not the central focus of our analysis, we also observed that fewer years of education, residing in a smaller locality size, owning fewer household goods and being single were negatively associated with mammography. (Supplementary Table 5).

Across all survey years the unadjusted prevalence was higher among respondents with IMSS or ISSSTE than among respondents without social security (Table 3). The largest differences were in 2015 (prevalence difference IMSS 24.0 % [95 % CI 16.8 %, 31.2 %]; ISSSTE 18.8 [9.5 %, 28.0 %]). Mammography prevalence was modestly lower among IMSS beneficiaries than among ISSSTE beneficiaries in survey waves 2001 (−6.1 % [−13.4, 1.2 %]) and 2003 (−6.5 % [−15.4 %, 2.4 %]). In later waves this pattern reversed; for instance, in 2012 mammography prevalence among was higher (8.2 % [−2.4 %, 18.8 %]) among IMSS beneficiaries than among ISSSTE beneficiaries. Of note, in 2018 there was a marked decline in mammography prevalence among ISSSTE beneficiaries relative to respondents with other insurance types (IMSS 16.7 % [7.8 %, 25.5 %]; no social security 6.3 % [−2.0 %, 14.7 %]). Adjustment for age, years education, household goods and marital status did not substantially change differences in prevalence between IMSS and ISSSTE beneficiaries but did substantially attenuate differences in prevalence between respondents with social security (IMSS and ISSSTE beneficiaries) and respondents without social security. Mammography prevalence increased substantially in all locality sizes between 2003 and 2012 but point estimates of these increases were larger in more urban areas (Supplementary Table 6).

**Table 3:** Difference in mammography prevalence between common insurance types by survey wave for women aged 50–69 in the Mexican Health and Aging Study, % (95 % CI).

	IMSS - ISSSTE		IMSS - No social security		ISSSTE - No social security	
Year	Unadjusted	Adjusted	Unadjusted	Adjusted	Unadjusted	Adjusted
2001	−6.1 (−13.4, 1.2)	−5.8 (−19.2, 7.6)	8.8 (4.8, 12.8)	4.7 (−3.2, 12.6)	14.9 (7.8, 21.9)	10.5 (−2.7, 23.7)
2003	−6.5 (−15.4, 2.4)	−7.3 (–23.0, 8.3)	10.2 (5.3, 15.0)	7.1 (−3.0, 17.1)	16.6 (8.1, 25.2)	14.4 (−0.7, 29.5)
2012	8.2 (−2.4, 18.8)	9.8 (−6.4, 26.0)	23.1 (15.9, 30.2)	12.7 (0.9, 24.5)	14.9 (5.1, 24.6)	2.9 (−13.7, 19.5)
2015	5.2 (−4.8, 15.2)	8.2 (−4.9, 21.4)	24.0 (16.8, 31.2)	12.3 (0.0, 24.6)	18.8 (9.5, 28.0)	4.1 (−10.8, 19.0)
2018	16.7 (7.8, 25.5)	17.9 (3.5, 32.4)	23.0 (16.9, 29.1)	12.7 (2.3, 23.1)	6.3 (−2.0, 14.7)	−5.2 (−20.4, 9.9)

Prevalence differences are differences between adjusted predictions at the mean estimated using Poisson generalized estimating equation with exchangeable correlation structure with survey year and insurance type as predictor and age in years, education level in years, number of household goods, marital status, locality size as covariates. The model included a significant interaction term between survey year and insurance type (Wald test; p = 0.002). IMSS = Instituto Mexicano de Seguro Social ISSSTE = Instituto de Seguridad y Servicios Sociales de los Trabajadores del Estado.

## 4 Discussion

This study evaluated national changes in mammography prevalence by insurance type from 2001 to 2018 among women in Mexico ages 50 to 69. It is the first study to assess changes in screening prevalence using the two-year prevalence interval that corresponds to national guidelines regarding screening frequency. We found that mammography prevalence increased substantially across all insurance types from 2003 to 2012 and experienced minimal change after this. However, disparities in screening persisted across the healthcare system with higher unadjusted screening rates observed across almost all survey waves among IMSS and ISSSTE beneficiaries when compared to respondents with no social security.

These results are discrepant with previously published reports of mammography prevalence in Mexico. Only one prior study has reported the two-year prevalence of mammography in Mexico using a dataset other than MHAS, finding a two-year prevalence of mammography of 29.4 % in women aged 50–69 in the 2012 wave of the *Encuesta Nacional de Salud y Nutrición* (ENSANUT) (Torres-Mejía et al., 2013). This is lower than the 2012 our two-year prevalence estimate of 56.5 % (53.2 %, 59.7 %) among women 50–69. Comparison between other years of MHAS and ENSANUT is challenging as ENSANUT only asks about the one-year prevalence of mammography in other years whereas MHAS consistently asks about the two-year prevalence. The same study cited above reports one-year mammography prevalence of 19.6 % and 22.8 % in the 2006 and 2012 waves of the ENSANUT survey for women age 50–69 (Torres-Mejía et al., 2013). A separate report using data from 2018 wave of ENSANUT reports a one-year mammography prevalence of 27.5 % in a broader age group (women aged 40–69). Several other reports provide similarly low estimates for one-year prevalence using data from a surveillance system run by the government, the *Sistema de Información en Cáncer de la Mujer* (SICAM*)* (Secretaria de Salud, 2012, Uscanga-Sánchez et al., 2014). While one-year and two-year prevalence estimates cannot be compared directly, this large difference also supports the existence of a discrepancy between mammography prevalence estimates from the present study and prior estimates of mammography prevalence in Mexico.

We considered possible reasons for the observed differences between our results and previous estimates of mammography prevalence. One explanation could be differences in exclusion criteria. In their study using data from ENSANUT 2012, Torres-Mejía et al. excluded women with breast cancer symptoms (the number of women excluded was not specified) while the present study did not as this information was not available in the MHAS (Torres-Mejía et al., 2013). Differences in question format could also contribute. When asking about two-year mammography prevalence in women aged 50–69, ENSANUT and MHAS used different question formats (Appendix A). A prior study examining the National Health Interview Survey in the United States found that asking about mammography using a one-part question, similar to the question used in the MHAS, led to a slightly higher reported prevalence than asking with a two-part question, like the question used in ENSANUT 2012 (Gonzales et al., 2017). Another possible reason for the discrepancies could be differences in the sample composition of ENSANUT and MHAS. However, this is unlikely as both surveys are nationally representative providing survey weights that expand to the total of the age eligible population of Mexico and lead to samples with similar socioeconomic characteristics (Torres-Mejía et al., 2013, Mexican Health and Aging Study, 2012, Wong et al., 2015, Romero-Martínez et al., 2012) Both the present study and Mejía-Torres et al used survey weights when estimating mammography prevalence (Torres-Mejía et al., 2013). A fourth explanation could be differential loss to follow up in the MHAS. However, restricting the analysis to first time MHAS respondents did not substantially affect the results suggesting that differential loss to follow did not have a significant impact. Other differences between the studies are unlikely to substantially contribute to differences in prevalence estimates (Appendix A). Harmonizing future surveys to ask about mammography prevalence using the two-year interval that corresponds to national guidelines for screening mammography could lead to estimates that are comparable and more relevant to health policy.

Although we found that mammography prevalence increased over the period studied, we also observed persistent disparities in screening between individuals with social security and without social security. Disparities attenuated after adjustment for education, locality size and household goods suggesting that these disparities may be partially due to differences in socioeconomic status. Women living in rural areas and with lower education levels had lower mammography prevalence and were less likely to have social security; this association has been described elsewhere as well (Agudelo, 2013, Balandrán-Duarte et al., 2020). Rurality and low socioeconomic status could impact access to screening as healthcare facilities are concentrated in urban areas and fees charged by private clinics for mammography may be prohibitively expensive. Although mammography prevalence increased substantially from 2003 to 2012 across all locality sizes, this increase was greater in more urban areas.

However, demographic characteristics included in our model may only partially explain observed disparities in screening between insurance types. Even after adjustment, in most survey years we found higher mammography prevalence in respondents with social security when compared to respondents without social security. These persistent differences could be due to health system factors such as differences in the accessibility of mammography across sectors of the healthcare system or differences in strategies for mammography outreach. More research is needed to better define the role of both socioeconomic factors and health system factors as contributors to disparities in mammography screening in Mexico.

### 4.1 Limitations

We note several limitations. Ascertainment of mammography prevalence relied on self-report. Women who have not participated in mammography might report doing so due to social desirability bias. The number of women excluded from our sample due to self-reported history of breast cancer is unexpectedly low when compared to estimates of breast cancer prevalence during this period which range from 0.75 % in 2001 to 0.98 % in 2018. (Global Burden of Disease Collaborative Network, 2019) This may be due to misclassification, however, as the absolute prevalence of breast cancer is low, this is unlikely to substantially impact our estimates of mammography prevalence. Our analysis did not allow us to distinguish between age, period, and cohort effects; further research is needed to assess the relative impact of each of these. Finally, publicly available datasets for the MHAS include survey weights, but do not include information about other aspects of survey design such as strata or sampling units so these could not be incorporated into our analysis. This would affect the reliability of confidence intervals but does not impact point estimates.

## 5 Conclusions

Ours was the first study to assess changes in mammography prevalence in Mexico using the two-year interval recommended by national screening guidelines. Our estimates showed a substantial increase in mammography prevalence between 2001 and 2012. Prevalence was relatively unchanged from 2012 to 2018 and disparities persisted between women with and without social security. More research is needed to confirm our findings regarding two-year mammography prevalence in Mexico and to better understand the causes of observed disparities.

## Funding

This project was partly supported by funding from the Global Cancer Program at the Helen Diller Family Comprehensive Cancer Center at the University of California San Francisco. The MHAS (Mexican Health and Aging Study) is partly sponsored by the National Institutes of Health/National Institute on Aging (Grant No NIH R01AG018016) in the United States and the Instituto Nacional de Estadística y Geografía (INEGI) in Mexico. SPM was supported by Health Resources and Services Administration (HRSA) grant (T32 HP 19025).

## CRediT authorship contribution statement

**Sean P. McClellan:** Conceptualization, Methodology, Formal analysis, Data curation, Writing – original draft, Writing – review & editing, Visualization. **Karla Unger-Saldaña:** Writing – review & editing. **John M. Neuhaus:** Formal analysis, Writing – review & editing. **Michael B. Potter:** Conceptualization, Writing – review & editing. **Carmen García-Peña:** Conceptualization, Writing – review & editing. **Jacqueline M. Torres:** Conceptualization, Writing – review & editing, Supervision.

## Declaration of Competing Interest

The authors declare that they have no known competing financial interests or personal relationships that could have appeared to influence the work reported in this paper.

## Data Availability

Data is public use and available at www.MHASweb.org. Code is available upon request.
