# Supplementary material for: Mammography prevalence in Mexico from 2001-2018: Results from the Mexican Health and Aging study
Source: Prev Med Rep. 2023 Feb 14;32:102150. doi: 10.1016/j.pmedr.2023.102150 (PMC9971542; doi:10.1016/j.pmedr.2023.102150)
Supplement: Supplementary data 1 [file mmc1.docx]

**Appendix A**

| Table A1 - Methodological aspects of the present study and Torres-Mejia et al. that might affect the comparability of prevalence estimates for the year 2012 including a comparison of the data sources used for each study^1^ | | |
| --- | --- | --- |
|  | **Present study** | **Mejía-Torres et al.**^1^ |
| **Comparison of the data sources used for each study** |  |  |
| Data source | Mexican Health and Aging Study (MHAS)^2^ | Encuesta Nacional de Salud y Nutrición (ENSANUT), 2012 wave^3^ |
| Agency responsible for survey design | Instituto Nacional de Estadística y Geografía (INEGI) | Instituto Nacional de Salud Pública (INSP) |
| Period of data collection | October 1 to November 23, 2012^4^ | October 3, 2011 to May 6, 2012^5^ |
| Design | Longitudinal national probability survey | Cross-sectional national probability survey |
| Population of interest | New respondents in 2012 survey wave: Individuals aged 50-60 in Mexico and their spouses, living in the community^4^  Follow up sample (recruited in in baseline survey wave in 2001): People aged 50 or older in 2001 in Mexico and their spouses, living in the community^6^ | Individuals of any age in Mexico, living in the community^5^ |
| Sampling frame | New respondents in 2012 survey wave: Selected from households where at least one member between age 50 and 60 was identified in the National Survey of Occupation and Employment conducted in 2012^4^  Follow-up sample. Includes respondents from the 2001 wave and their spouses. Respondents in the 2001 survey were selected from households where at least one individual age 50 or older was identified in the National Occupation Survey conducted in 2000.^6^ | Households were selected from the 2005 *Conteo de Población y Vivienda* and the list of new communities identified in the 2010 census.^5^ |
| Proxy interviews | For respondents unable to answer survey questions, including deceased respondents, proxy interviews were conducted with other members of the household. However, proxy interviews were excluded from the present study.^4^ | Methodological documents do not discuss proxy interviews.^5,7^ |
| Survey weights | For new respondents in the 2012 wave of the MHAS, the methodological document states survey weights were calculated  based on the inverse probability of a respondent being selected to participate in the survey and adjusted for non-response.^7^  For the follow-up sample, the methodological document from the 2001 wave of the MHAS survey notes that the dataset includes individual weights designed to expand to the population of adults age and older in Mexico but does not provide a detailed description of how weights were calculated.^6^ | The 2012 ENSANUT dataset includes both household and survey weights. A published summary of the study’s design notes that these weights considered intentional oversampling of households with high levels of marginalization.^7^ |
| Survey question inquiring about mammography prevalence | 1 part question (see below for full question) | 2-part question (see below for full question) |
| **Comparison of the methods used for each study** |  |  |
| Inclusion criteria | Women aged 50-69 | Women aged 40-49 and aged 50-69. |
| Exclusion criteria | Proxy interviews and interviews in which women endorsed a history of breast cancer | Women with history of breast cancer or signs or symptoms of breast cancer. |
| Missing values | Observations with missing screening or covariate data were excluded | Not specified in article |
| Use of survey weights | Non-standardized survey weights were used (weights expand to the total of the age eligible population in Mexico) | The article states that analysis was conducted considering the sample design of the survey. |

**Mammography questions from the Mexican Health and Aging Survey (MHAS) 2012 and the Encuesta Nacional de Salud y Nutrición 2012**

For the 2012 wave of the Encuesta Nacional de Salud y Nutrición (ENSANUT) the interview guide instructs the interviewer to ask^8^:

“*Alguna vez en su vida, ¿un médico o enfermera, le ha realizado una mastografía?”*

“At any time in your life, has a doctor or a nurse given you a mammogram?” (author’s translation)

If the respondent answers yes, the interviewer is instructed to ask the following questions:

*“Durante el 2011, ¿acudió al módulo de medicina preventiva para mastografía?*

*Durante el 2010, ¿acudió al módulo de medicina preventiva para mastografía?*

*Durante el 2009, ¿acudió al módulo de medicina preventiva para mastografía?*

*¿Hace cuánto tiempo le hicieron su última mastografía?”*

“During 2011, did you go to the preventive medicine module for a mammogram?

During 2010, did you go to the preventive medicine module for a mammogram?

During 2009, did you go to the preventive medicine module for a mammogram?

When was the last time you were given a mammogram?”

(author’s translation)

For the 2012 wave of the Mexican Health and Aging Survey (MHAS) the interview guide instructs the interviewer to ask^2^:

*“En los últimos dos años ¿ha tenido alguna de las siguientes pruebas o procedimientos médicos?... ¿Le han hecho una mamografía o una radiografía de los senos para detectar cáncer?*

In the last two years, have you had any of the following exams or medical procedures?... Have you had a mammogram or x-ray to check for breast

cancer? (from English version of interview guide)

**References**

1. Torres-Mejía G, Ortega-Olvera C, Ángeles-Llerenas A, Villalobos-Hernández AL, Salmerón-Castro J, Lazcano-Ponce E, Hernández-Ávila M. Utilization patterns of prevention and early diagnosis for cancer in women. Salud Publica Mex. 2013;55 Suppl 2:S241-8. PMID: 24626701

2. Mexican Health and Aging Study. MHAS, Mexican Health and Aging Study, 2001-2018. Data Files and Documentation (public use) [Internet]. [cited 2022 Jan 20]. Available from: www.MHASweb.org

3. Instituto Nacional de Salud Pública, México. ENSANUT (Encuesta Nacional de Salud y Nutrición) 2012 [Internet]. Available from: https://ensanut.insp.mx/encuestas/ensanut2012/descargas.php

4. Mexican Health and Aging Study 2012: Sample design [Internet]. Instituto Nacional de Estadística y Geografía; 2013. Available from: http://mhasweb.org/Resources/DOCUMENTS/2012/Methodological_Document_2012%E2%80%93SEC.pdf

5. Encuesta Nacional de Salud y Nutrición 2012. Resultados nacionales [Internet]. Instituto Nacional de Salud Pública; 2012. Available from: https://ensanut.insp.mx/encuestas/ensanut2012/doctos/informes/ENSANUT2012ResultadosNacionales.pdf

6. Instituto Nacional de EstadÌstica, GeografÌa e Informática. Estudio Nacional de Salud y Envejecimiento en México (ENASEM) 2001: Documento Metodológico Reporte de Proyecto. 2004 Jun;

7. Romero-Martínez M, Shamah-Levy T, Franco-Núñez A. Encuesta Nacional de Salud y Nutrición 2012: diseño y cobertura. Salud Pública de México. saludpublica.mx; 2013;55:S332–S340.

8. Instituto Nacional de Salud Pública, México. ENSANUT (Encuesta Nacional de Salud y Nutrición) 2018 [Internet]. Available from: https://ensanut.insp.mx/encuestas/ensanut2018/descargas.php
